# Disease-associated variants in different categories of disease located in distinct regulatory elements

**DOI:** 10.1186/1471-2164-16-S8-S3

**Published:** 2015-06-18

**Authors:** Meng Ma, Ying Ru, Ling-Shiang Chuang, Nai-Yun Hsu, Li-Song Shi, Jörg Hakenberg, Wei-Yi Cheng, Andrew Uzilov, Wei Ding, Benjamin S Glicksberg, Rong Chen

**Affiliations:** 1 Department of Genetics and Genomic Sciences, Icahn School of Medicine at Mount Sinai, New York, NY, 10029, USA; 2 Department of Neuroscience, Icahn School of Medicine at Mount Sinai, New York, NY, 10029, USA; 3 Department of Endocrinology, Anhui Provincial Hospital, Hefei, Anhui, 230001, China; 4 School of Computer Science and Technology, Anhui University, Hefei, Anhui, 230039, China

**Keywords:** disease-associated variants, regulatory elements, recurrent cancer somatic mutation, cancer predisposing germline variant, Mendelian disease, complex disease, promoter, histone modification, chromatin physical interaction

## Abstract

**Background:**

The invention of high throughput sequencing technologies has led to the discoveries of hundreds of thousands of genetic variants associated with thousands of human diseases. Many of these genetic variants are located outside the protein coding regions, and as such, it is challenging to interpret the function of these genetic variants by traditional genetic approaches. Recent genome-wide functional genomics studies, such as FANTOM5 and ENCODE have uncovered a large number of regulatory elements across hundreds of different tissues or cell lines in the human genome. These findings provide an opportunity to study the interaction between regulatory elements and disease-associated genetic variants. Identifying these diseased-related regulatory elements will shed light on understanding the mechanisms of how these variants regulate gene expression and ultimately result in disease formation and progression.

**Results:**

In this study, we curated and categorized 27,558 Mendelian disease variants, 20,964 complex disease variants, 5,809 cancer predisposing germline variants, and 43,364 recurrent cancer somatic mutations. Compared against nine different types of regulatory regions from FANTOM5 and ENCODE projects, we found that different types of disease variants show distinctive propensity for particular regulatory elements. Mendelian disease variants and recurrent cancer somatic mutations are 22-fold and 10- fold significantly enriched in promoter regions respectively (q<0.001), compared with allele-frequency-matched genomic background. Separate from these two categories, cancer predisposing germline variants are 27-fold enriched in histone modification regions (q<0.001), 10-fold enriched in chromatin physical interaction regions (q<0.001), and 6-fold enriched in transcription promoters (q<0.001). Furthermore, Mendelian disease variants and recurrent cancer somatic mutations share very similar distribution across types of functional effects.

We further found that regulatory regions are located within over 50% coding exon regions. Transcription promoters, methylation regions, and transcription insulators have the highest density of disease variants, with 472, 239, and 72 disease variants per one million base pairs, respectively.

**Conclusions:**

Disease-associated variants in different disease categories are preferentially located in particular regulatory elements. These results will be useful for an overall understanding about the differences among the pathogenic mechanisms of various disease-associated variants.

## Background

Along with the wide application of high throughput technologies, hundreds of millions genetic variants have been identified with a dramatic growth of dbSNP occurring after 2007 [1]. From these resources/studies, it was found that ~97% of all identified variants are noncoding variants, consistent with the notion that 98% of human genome sequences are noncoding [2]. The studies that have resulted from the ENCODE project show that over 80% of human genome are functional [3], participating in at least one biochemical RNA- or chromatin-associated event in at least one cell type. Any variant that is located within a functional genomic region potentially has the ability to cause a dysregulation on gene expression through modifying regulatory elements, possibly resulting in diseases pathogenesis [4,5]. A lot of well-annotated disease-variants have been collected in the Human Gene Mutation Database (HGMD) [6]; these variants are organized into three groups of significant functional disease SNPs, namely coding SNPs (cSNPs), splicing SNPs (sSNPs) and regulatory SNPs (rSNPs), which account for ~86%, ~10% and ~3% of variants in HGMD respectively [6-9]. There is plenty of information about coding variants but limited knowledge about noncoding variants. In recent years, genome-wide association studies (GWAS) [10] identified over ten thousand variants associated with various diseases/traits, ~90% of which localize outside of known protein-coding regions. This phenomenon highlights the substantial gap between the plethora of disease- or trait-associated noncoding variants and our understanding of how most of these variants contribute to diseases/traits. (Figure S1)

Gene expression is a tightly regulated process, involving various regulatory elements including promoters, enhancers, insulators, and silencers. Moreover, the chemical modifications (i.e. methylation and acetylation) on histone proteins present in chromatin has been shown to change the accessibility of the chromatin for transcription to occur and thusly influence gene expression [11,12]. Some projects, such as ENCODE [3] and FANTOM5 [13,14], adopted various experimental technologies including ChIP- seq [15], DNase-seq [16], ChIA-PET [17], and CAGE [18-21], and identified a lot of various regulatory regions throughout the human genome across hundreds of tissues and cell types [22]. These various experiments validated regulatory regions datum provide an opportunity to investigate the underlying pathogenic mechanism of disease-associated variants.

A possible mechanism underlying the pathogenesis of disease-associated variants is the disruption of the binding of transcription factors, local chromatin structure, and/or co-factors recruitment, ultimately altering the expression of the target genes. Some published studies support such a hypothesis through analyzing the distribution of regulatory complex disease variants by GWAS [3,23-30]. In the current study, we focus on the dissimilarity of underlying pathogenic regulatory mechanisms of disease-associated variants in different disease categories, including Mendelian diseases, complex diseases, cancer predisposing germline variants, and recurrent cancer somatic mutations.

## Results and discussion

### Distinct densities of disease-associated variants within different types of regulatory regions

#### Curation of disease-associated variants and regulatory regions

We curated disease-associated variants for Mendelian diseases, germline cancers, somatic cancers, and complex diseases (see details in supplementary materials, Additional File 1). Disease-associated variants are summarized in Table 1. There were 27,558 Mendelian disease variants collected from OMIM [31] and ClinVar [32], residing within 2,229 genes and causing 5,317 diseases/phenotypes. VarDi is a database of disease-associated variants built through a combination of Hadoop-based text mining tools and manual curation [33]. We collected 20,964 complex disease variants from VarDi and NHGRI GWAS Catalog [10], located within 2,615 genes and associated with 1,243 diseases/traits. Compared with 5,809 cancer predisposing germline variants from HGMD professional database [6], 43,364 recurrent cancer somatic mutations were extracted from COSMIC [34]. Cancer predisposing germline variants were located across 294 genes, while recurrent cancer somatic mutations were distributed throughout 14,649 genes.

**Table 1:** Summary of disease variants.

Variants	Data sources	#Variants	#Genes	#Diseases/Phenotypes/Traits
Mendelian disease variants	OMIM, ClinVar	27,558	2,229	5,317
Complex disease variants	GWAS catalog, VarDi	20,964	2,615	1,243
Cancer predisposing germline variants	HGMD professional	5,809	294	260
Recurrent cancer somatic mutations	COSMIC	43,364	14,649	296

A lot of regulatory regions, including transcription promoters, enhancers and insulators, DNA methylation regions, histone modification regions, chromatin physical interaction regions, DNA binding sites of protein factors by ChIP-seq, and open chromatin regions by DNase-seq and FAIRE-seq, were identified by the FANTOM5 and ENCODE projects, which are summarized in Table 2. Transcription promoter, enhancer, and insulator regions account for 0.12%, 0.38% and 3.52% of the human genome respectively. Roughly 0.6% of the human genome is DNA methylation regions, usually overlapping with transcription promoter regions. Histone modification regions occupy over 87% of the human genome, revealing the ubiquity of epigenetic marked regions. About 40% of the human genome involves chromatin physical interaction zones, hinting an abundant, long-range regulation during gene expression process. Similar percentages of human genome, 11.76%, 11.97% and 13.87%, comprise DNA binding sites of protein by ChIP-seq and open chromatin regions by DNase-seq and FAIRE-seq, respectively.

**Table 2:** Summary of regulatory regions from FANTOM5 and ENCODE.

Regulatory Regions	Source	Technique	Institute	Length(bp)	Percent of human genome (%)
Transcription promoter	FANTOM5	CAGE	RIKEN	3,833,500	0.12
Transcription enhancer	FANTOM5	CAGE	RIKEN	12,385,403	0.38
Transcription insulator	ENCODE	ChIP-seq	HudsonAlpha Institute for Biotechnology, Yale University, Harvard University	81,713,060	3.52
Methylation region	ENCODE	Methylation 450	HudsonAlpha Institute for Biotechnology	19,517,834	0.60
Histone modification region	ENCODE	ChIP-seq	Broad institute, Massachusetts General Hospital, Harvard Medical School	2,816,878,674	
Chromatin physical interaction regions	ENCODE	CHIA-PET	Genome Institute of Singapore, Stanford University	1,288,430,643	39.83
DNA binding sites of protein	ENCODE	ChIP-seq	HudsonAlpha Institute for Biotechnology, Yale University, Harvard University	380,355,257	11.76
Open chromatin regions (DNase I hypersensitive sites)	ENCODE	DNase-seq	Washington University, Duke University	387,138,495	11.97
Open chromatin regions by FAIRE-seq	ENCODE	FAIRE-seq	Duke University, University of North Carolina at Chapel Hill, University of Texas at Austin, European Bioinformatics Institute, University of Cambridge	448,557,442	13.87

#### Regulatory regions are widely located within coding and noncoding regions

We extracted seven types of human genomic regions: coding exons, 5'-UTR, 3'-UTR, introns, upstream and downstream 2000bp of genes, and intergenic region; and then counted the regulatory regions overlapping with each type of the genomic regions (Table 3, **S1**). Regulatory regions are widely located within different human genomic regions, and each type of human genomic regions also can contain various regulatory regions. Transcription promoters can occur within each type of genomic region. ~45% of promoters can be within intergenic regions, hinting a lot of potential protein or RNA genes unknown in the intergenic regions. Intronic promoters account for ~24%, consistent with the study that lots of latent noncoding RNA genes within introns [35]; Intronic promoters are associated with various disorders, including cancer [36]. Dr. Ingolia found pervasive translation outside of annotated protein-coding genes through ribosome profiling analysis, implying that a lot of transcription promoters are located outside of protein-coding genes [37]. Transcription enhancers and insulators mainly locate within intergenic regions, introns, upstream and downstream of genes. Over 15% of methylation regions occurs within coding exons, 5'-UTR and upstream of genes. The majority of regulatory regions are located within noncoding regions including introns and intergenic regions, while coding regions also contain various regulatory regions. 54.99% of coding exons are overlapped with regulatory regions, implying the regulatory role of coding exons on gene expression. ~15% of coding exon regions can be DNA binding sites of proteins, which is in agreement with the study that genetic code specifying amino acids and regulatory code specifying transcription factor recognition sequences has been proven to exist simultaneously within human protein coding regions [38]. Regulatory activity on gene expression can occur within any type of human genomic regions.

**Table 3:** Percentage of each types of regulatory regions overlapped with different human genomic regions

	Overlapping Coding Exon (bp)	Overlapping Upstream (bp)	Overlapping 3'-UTR (bp)	Overlapping 5'-UTR (bp)	Overlapping Introns (bp)	Overlapping Downstream (bp)	Overlapping Intergenic Regions (bp)
Transcription	318,864	1,033,885	235,904	793,623	948,521	202,588	1,747,482
promoter	(8.32%)	(26.97%)	(6.15%)	(20.7%)	(24.74%)	(5.23%)	(45.58%)
Transcription	275	443,003	12,826	8,301	4,351,818	326,034	7,628,892
enhancer	(0.002%)	(3.58%)	(0.1%)	(0.07%)	(35.14%)	(2.63%)	(61.6%)
Transcription	1,678,883	6,568,355	1,642,952	2,007,230	25,080,619	3,312,161	49,525,266
insulator	(2%)	(8.04%)	(2%)	(2.45%)	(30.7%)	(4.05%)	(60.6%)
Methylation	961,891	3,066,144	790,094	850,738	5,552,194	1,039,107	10,745,614
region	(4.93%)	(15.71%)	(4.04%)	(4.36%)	(28.45%)	(5.32%)	(55%)
Histone	19,836,913	65,289,808	27,693,911	13,261,068	901,286,244	69,178,945	1,820,842,829
modification region	(0.7%)	(2.32%)	(1%)	(0.47%)	(32%)	(2.46%)	(64.64%)
Chromatin	14,486,785	45,832,380	20,066,412	9,708,604	464,537,271	46,072,176	761,585,471
physical interaction regions	(1.12%)	(3.56%)	(1.6%)	(0.75%)	(36.05%)	(3.58%)	(59.1%)
DNA binding	5,252,509	22,329,204	6,553,411	5,567,759	130,492,231	14,945,226	224,621,692
sites of protein	(1.38%)	(5.87%)	(1.7%)	(1.46%)	(34.3%)	(3.93%)	(59.06%)
Open	6,309,327	22,046,177	6,966,217	5,626,301	131,734,716	15,003,875	229,306,915
chromatin regions (DNase	(1.63%)	(5.69%)	(1.8%)	(1.45%)	(34.02%)	(3.88%)	(59.23%)
I hypersensitive sites)							
Open	4,896,207	18,119,824	6,098,267	4,612,397	137,079,859	12,725,401	290,428,922
chromatin regions by	(1.09%)	(4.03%)	(1.4%)	(1.02%)	(30.56%)	(2.84%)	(64.75%)
FAIRE-seq							

Illumina SureSelect TruSeq and Nimblegen SeqCap EZ are two popular exome DNA sequencing technologies which can be used to identify Mendelian disease variants, cancer predisposing germline mutations and cancer somatic mutations. The target regions of these two exome DNA sequencing platforms can be located within various human genomic regions (Table S2, S3). Moreover, these target regions also are overlapped with various regulatory regions (Table S4, S5), suggesting any disease variants identified by such exome DNA sequencing platform can likely be located within any type of regulatory regions.

#### Highest density of disease-associated variants within transcription promoter

We counted the number of disease-associated variants within each type of regulatory regions, and calculated the average number of **d**isease **v**ariants **p**er one **m**illion base pairs of regulatory regions (DVPM) (Table 4). The highest density of disease-associated variants was in transcription promoter (472 DVPM), which is reasonable considering the importance of transcription promoters in initiating gene expression. Methylated regions had a DVPM of 239, which usually overlap with transcription promoter regions. Transcription insulator had the third highest density of 72 DVPM, while transcription enhancer region had the lowest DVPM 0f 18. Transcription insulator regions are tightly associated with the 3D structure of DNA, mediated by CTCF protein. Accordingly, variants in insulator regions can result in changes of the 3D structure of DNA [39]. The density of disease variants in other types regulatory regions range from 33 to 68. The disease-associated variants have quite different densities in various regulatory regions.

**Table 4:** Summary of disease variants residing within regulatory regions in seven types of human genomic regions

	#Disease Variants within upstream	#Disease Variants within 5'UTR	#Disease Variants within coding exons	#Disease Variants within introns	#Disease Variants within 3'UTR	#Disease Variants within downstrea m	#Disease Variants within intergenic region	Total (unique variants)	DVPM
Transcription promoter	666	589	1,675	480	555	691	7	1,812	472
Methylation region	1,378	1,062	4,113	1,440	1,196	1,317	48	4,671	239
Transcription insulator	1,876	1,472	4,637	1,976	1,393	1,790	279	5,886	72
Open chromatin regions (DNase I hypersensitiv e sites)	6,661	5,776	20,306	9,580	7,334	7,336	1214	26,188	68
DNA binding sites of protein	6,360	5,122	17,211	9,043	6,181	7,016	1143	22,915	60
Chromatin physical interaction regions	13,775	13,040	49,007	26,125	19,005	17,952	3447	65,309	51
Open chromatin regions by FAIRE-seq	5,612	5,013	16,922	8,779	6,434	6,261	1203	22,470	50
Histone modification region	16,862	16,783	69,303	35,889	24,566	22,090	6196	93,212	33
Transcription enhancer	30	1	0	129	1	12	71	219	18

DVPM: the average number of disease variants per one million base pairs regulatory regions.

### Similar pattern of functional effects between Mendelian disease variants and recurrent cancer somatic mutations

**Figure 1:**
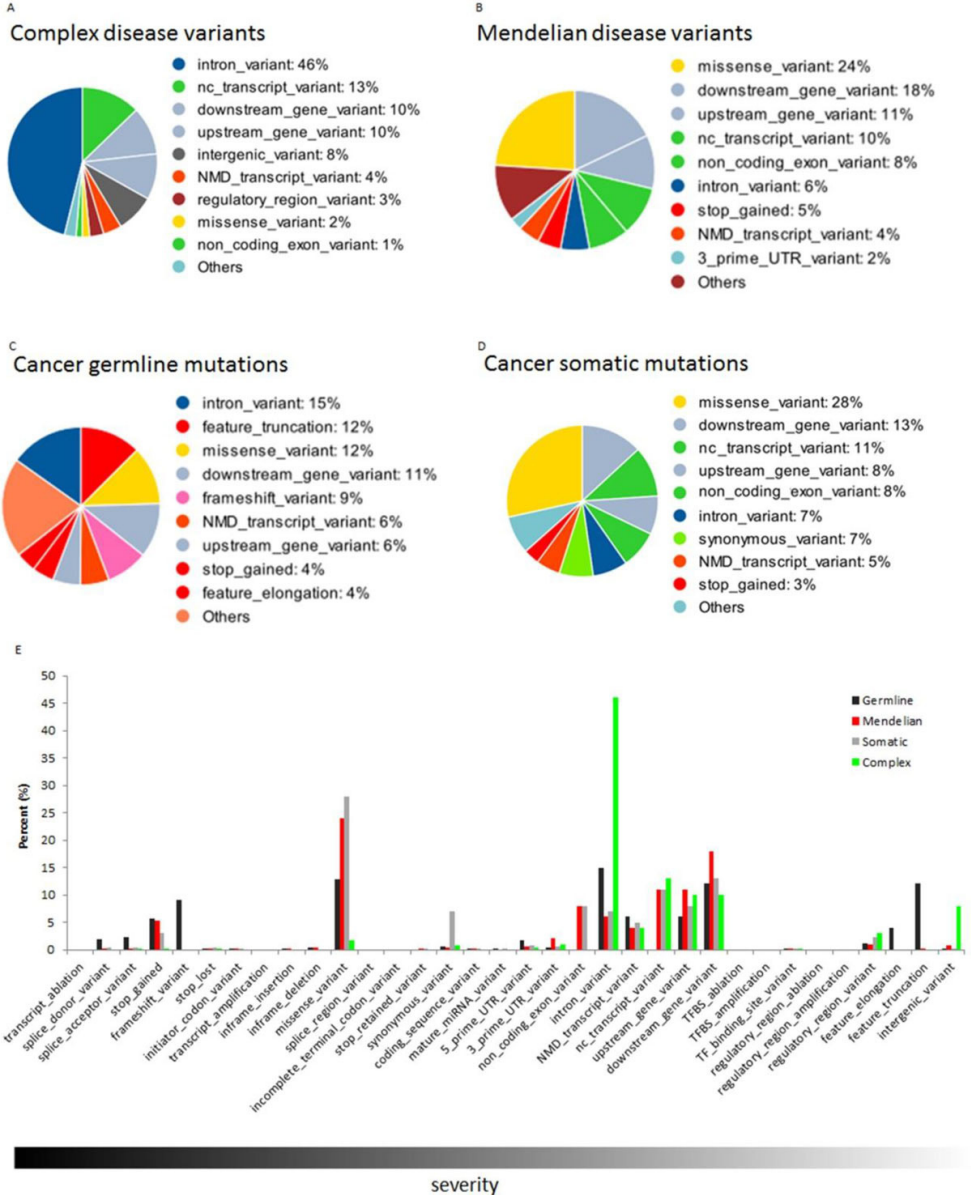
**Functional annotation of four types of disease associated variants**. (A), (B), (C) and (D) are the annotation results for Complex disease variants, Mendelian disease variants, Cancer predisposing germline mutations and Recurrent cancer somatic mutations using Ensembl Variants Effect Predictor respectively. Majority of complex disease variants are noncoding variants. Mendelian disease variants and recurrent cancer somatic mutations share similar pattern of functional effects. Compared with complex disease variants, more other three types of disease variants locate within coding region. (E) The histogram for the distribution of consequences of the four types of disease variants. The consequences by Mendelian disease variants, cancer predisposing germline variants and recurrent cancer somatic mutations are more serious than that of complex disease variants.

We applied Ensebml Variants Effect Predictor [40] to annotate the functional effects of disease variants in four disease categories (Figure 1A-D). Functional effects of variants can be classified into 34 consequences in Sequence Ontology [41], which were ranked in the order of severity (more severe to less severe) by Ensembl analysis group [40,42] (Figure 1E, Table S6). Mendelian disease variants and recurrent cancer somatic mutations share the same top functional effects: missense_variant (24% vs 28%), downstream_gene_variant (18% vs 13%), upstream_gene_variant (11% vs 8%), nc_transcript_variant (10% vs 11%), non_coding_exon_variant (8% vs 8%), intron_variant (6% vs 7%), NMD_transcript_variant (4% vs 5%) and stop_gained (5% vs 3%). Mendelian disease variants and recurrent cancer somatic mutations show similar pattern of functional effects.

The majority of complex disease variants are noncoding variants. Intron_variant (46%), upstream_gene_variant (10%), downstream_gene_variant (10%) and intergenic_variant (8%) sum up to ~75% of the overall complex disease variants. Considering that complex disease variants identified via GWAS are not necessarily the causal variants, and functional annotation of the GWAS SNPs may not reflect the nature of complex disease causal variants, we further recompiled the annotation on those complex disease variants that were replicated in at least two different ethnicities, and more likely to be causal than just markers. We produced a similar annotation result for complex disease causal variants (Figure S2). Intron_variant (39%), upstream_gene_variant (19%), downstream_gene_variant (19%), and intergenic_variant (4%), sum up to ~80% of the overall complex disease causal variants, supporting complex disease causal variants mainly are located within noncoding region.

More deleterious functional effects are found for Mendelian disease variants, cancer predisposing germline variants, and recurrent cancer somatic mutations compared to complex disease variants. Deleterious functional effects, such as stop_gained and frameshift_variant make up a substantial part of recurrent cancer somatic mutations, cancer predisposing germline variants and Mendelian disease variants. We generated a histogram of the functional effects of the four types of disease variants (Figure 1E). Roughly 5% of cancer predisposing germline variants change splice sites, suggesting abnormal splicing isoforms caused by variants might lead to cancer formation. Stop_gained variants may result in a prematurely ended protein product, which is notable among the consequences of Mendelian disease variants, cancer predisposing germline variants and recurrent cancer somatic mutations. The top eleven serious consequences, specifically transcript_ablation, splice_donor_variant, splice_acceptor_variant, stop_gained, frameshift_variant, stop_lost, initiator_codon_variant, transcript_amplication, inframe_insertion, inframe_deletion and missense_variant (Figure 1E, Table S4), account for 32.39%, 31.95%, 30.55% and 1.8% in cancer predisposing germline variants, recurrent cancer somatic mutations, Mendelian disease variants, and complex disease variants respectively. The majority of complex disease variants were annotated by the bottom fifteen consequences categories, suggesting milder functional effect of complex disease variants compared to other three types of disease variants. Accordingly, cancer predisposing germline variants, recurrent cancer somatic mutations, and Mendelian disease variants tend to cause more serious consequences compared to complex disease variants.

A limitation of this analysis is that the SNPs, which are linkage disequilibrium with complex disease variants, were not considered for the functional effect annotation analysis. Even so, we still accept that the complex disease associated variants can reflect the main properties of the disease-associated linkage disequilibrium genomic regions where the complex disease causal variants may locate. Therefore, this functional effect annotation analysis here can be helpful to understand the dissimilarity among the functional effects of the four types of disease-associated variants.

### Positive correlation between functionality of disease variants and evolutionary constraints on the disease variants

A series of bioinformatics tools have been developed to predict whether variants are functional or deleterious. We applied GWAVA [43], Mutation Assessor [44], CADD[45], and GERP [46,47] to score and measure the functionality of the four types of disease variants.

**Figure 2:**
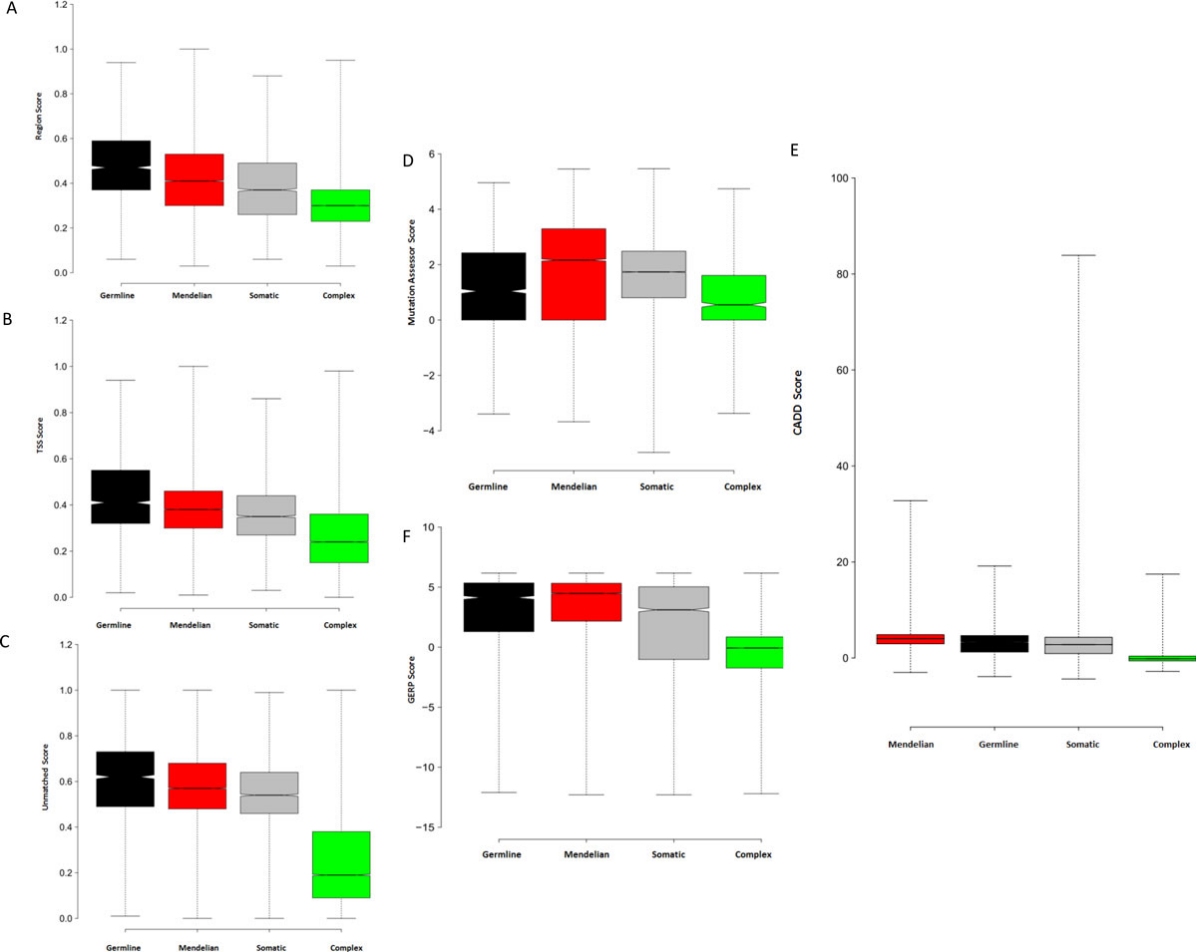
**Prediction scores for the disease associated variants**. Disease associated variants were annotated by GWAVA (A, B, C), Mutation Assessor (D), CADD (E) and GERP (F). GWAVA score noncoding variants. Mutation Assessor score coding variants. CADD score coding or noncoding variants. GERP estimates the evolutionary constraints on genomic site. There are there types of GWAVA score: Region Score (A), TSS Score (B) and Unmatched Score (C). GWAVA annotation results indicate that the functionality of noncoding disease variants degrade in the order of cancer predisposing germline variants, Mendelian disease variants, recurrent cancer somatic mutations and complex disease variants. (D) Mutation Assessor annotation result shows that more cancer predisposing germline variants, Mendelian disease variants and recurrent cancer somatic mutations are with at least medium functionality compared to complex disease variants. (E) CADD annotation result suggests that deleteriousness of disease variants essentially decrease in the order of Mendelian disease variants, cancer predisposing germline variants, recurrent cancer somatic mutations and complex disease variants. (F) GERP annotation result indicates that the evolutionary constraints on disease variants are positively correlated with the functionality of disease variants, namely, the greater the functionality of disease variants, the greater the evolutionary constraints.

The functionalities of cancer predisposing germline variants, Mendelian disease variants, and recurrent cancer somatic mutations are greater than that of complex disease variants. GWAVA aims to predict functionality of noncoding variants. There are three kinds GWAVA scores: Region score, TSS score, and Unmatched score. A high GWAVA score means more active functionality with respect to a low GWAVA score. On the whole, the functionality of noncoding disease variants degrades in the order of germline cancer, Mendelian disease, somatic cancer, and complex disease (Figure 2A,B,C). Mutation Assessor predicts the functional impact of coding variants on the protein level. Usually functional coding variants have a higher Mutation Assessor score than non-functional coding variants. Mutation Assessor score threshold of 1.9 was used to discriminate disease-associated variants with medium or high functionality [44]. Within all the coding disease variants, we found that 55.31% of Mendelian disease variants, 44.65% of recurrent cancer somatic mutations, 36.2% of cancer predisposing germline variants, and 16.69% of complex disease variants are with medium or high functionality (Figure 2D). In general, the associated coding variants for germline cancer, Mendelian disease and somatic cancer are more functional than the associated coding variants for complex disease. CADD integrates multiple annotations to score the deleteriousness of coding or noncoding variants in the human genome. A high CADD score typically suggests more sever deleteriousness compared to a low CADD score. Some recurrent cancer somatic mutations have a very high CADD score, implying exceptional deleteriousness. By and large, however, Mendelian disease variants are the most deleterious. Complex disease variants in comparison are mild. Some recurrent cancer somatic mutations have a low, negative CADD score, and as such, are most likely neutral (Figure 2E). Overall, the deleteriousness of disease variants gradually increases in the order: complex disease variants, recurrent cancer somatic mutations, cancer predisposing germline variants, and Mendelian disease variants. The prediction score annotations for the four types of disease variants by GWAVA, Mutation Assessor, and CADD all suggest that cancer predisposing germline variants, Mendelian disease variants and recurrent cancer somatic mutations are more functional than complex disease variants.

Functional disease-associated variant is prone to under the evolutionary constraint. GERP [46,47] can produce position-specific estimates of evolutionary constraint. Negative GERP scores indicate that a site is most likely evolutionary neutral. Positive scores suggest that a site may be under evolutionary constraint. Positive scores scale with the level of constraint, such that the greater the score, the greater the level of evolutionary constraint on that site. We found that 82.41% of cancer predisposing germline variants, 86.06% of Mendelian disease variants, 70.22% of recurrent cancer somatic mutations have a positive GERP score, while ~60% of complex disease variants have a negative GERP score (Figure 2F), indicating that variants in the former group are under evolutionary constraint, while the majority of complex disease variants are evolutionary neutrally. Moreover, GWAVA, Mutation Assessor and CADD annotations of the four types of disease variants all suggest that the functionality of cancer predisposing germline variants, Mendelian disease variants, and recurrent cancer somatic mutations is greater than that of complex disease variants. By and large, the GERP score of the disease variants gradually decrease in the order of Mendelian disease variants, cancer predisposing germline variants, recurrent cancer somatic mutations, and complex disease variants. Thus, the aforementioned observations rationally lead to the conclusion that the greater the functionality of the disease variant, the greater the level of evolutionary constraint.

### Disease-associated variants in different disease categories are located within particular regulatory regions

**Figure 3:**
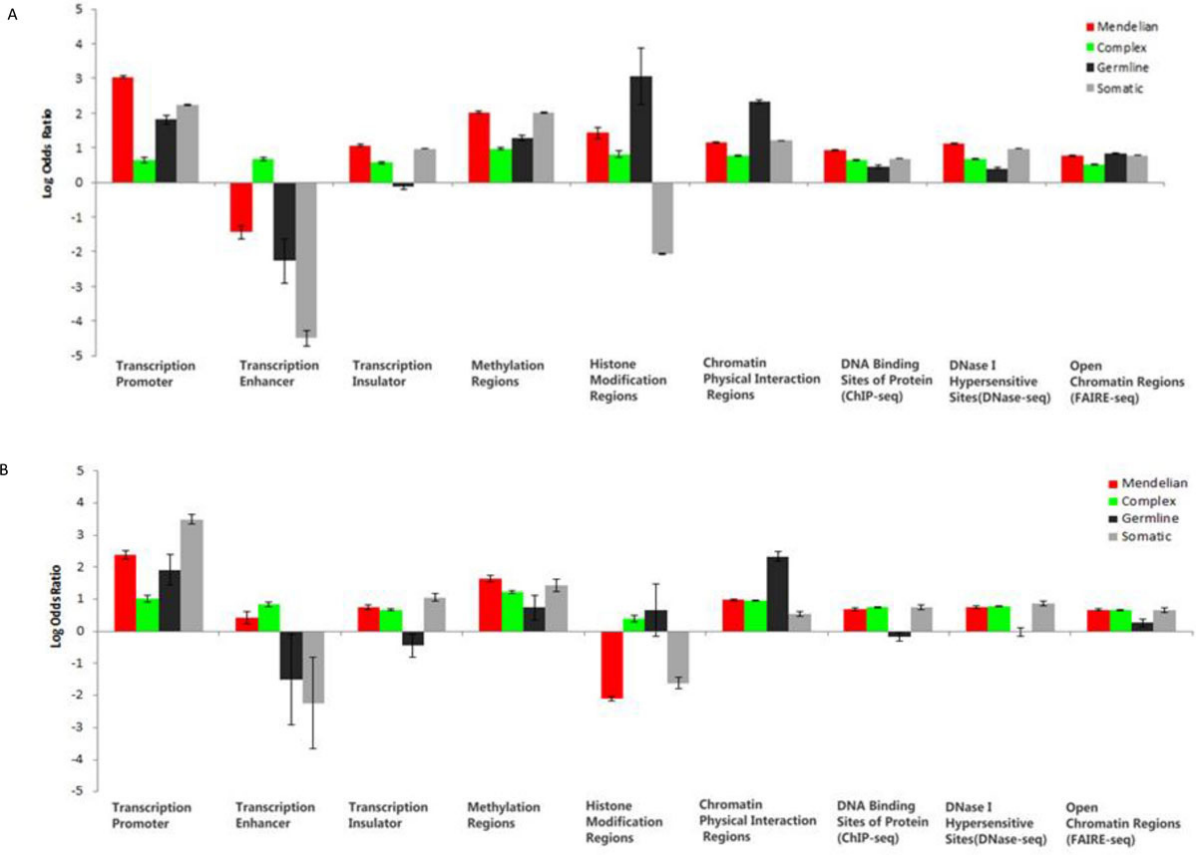
**Enrichment analysis of disease variants wihtin regulatory regions**. (A) Enrichment analysis of disease vairants to human genomic variants. Compared with human genomic variant background (control group), the natural logorithm of odds ratio of disease variants to control group was calculated. The error bar means standard error. Overall, diseaase variants are enriched within regulatory regions. Moreover, different types of disease variants show distinctive propensity for particular regulatory elements. Transcription promoter are the most enriched regulatory regions for Mendelian disease variants and recurrent cancer somatic mutations. Cancer predisposing germline variants are over ten times enriched within histon modification regions and chromatin physical interaction regions. Complex disease variants show quite even enrichment distribution within various regulatory regions. (B) Enrichment analysis of noncoding disease variants to human genomic noncoding SNPs. Disease variants and noncoding disease variants show similar enrichment pattern within various regulatory regions. Recurrent cancer somatic noncoding variants and Mendelian disease noncoding variants are most enriched within transcription promoter. Cancer predisposing germline noncoding variants are most enriched within chromatin physicial interaction regions. Complex disease noncoding variants are with quite even enrichment wihtin different regulatory regions.

There is a pressing need to understand the pathogenic mechanism of disease-associated variants along with the wide application of high throughput sequencing technologies. Disease-associated variants located within regulatory regions, which cause dysregulation of the gene expression process, and result in abnormal protein products, is an important and efficient pathogenic mechanism. Therefore, the disease variants should be enriched within regulatory regions when compared to a control human genome variant background. The human genome variant background was generated as control group by subtracting four types of disease-associated variant from all SNPs that appear in the dbSNP database. We applied an odds ratio to measure the enrichment of disease variants within regulatory regions. We then plotted the natural logarithm of the odds ratios of disease variants to the control genome variant background within various regulatory regions (Figure 3A), which were tested statistically using a Pearson chi-squared test (Table S7).

Overall, the enrichments of different types of disease variants within various regulatory regions are different from each other. Enrichment of Mendelian disease variants, recurrent cancer somatic mutations, and cancer predisposing germline variants within transcription promoter regions are 21 times (log value 3.04), 10.57 times (log value 2.36) and 6.1 times (log value 1.8) higher than that of the genome variant background respectively, in contrast to only 1.9 times (log value 0.64) for complex disease variants. This implies that transcription promoters might be an efficient mechanism for Mendelian disease and cancer (germline or somatic), but not for complex disease pathogenesis. Additionally, the enrichment profile of the four types of disease variants within methylation regions just like that within transcription promoters. Mendelian disease variants, recurrent cancer somatic mutations, and complex disease variants show higher enrichment within transcription insulator regions than cancer predisposing germline variants. Most disease variants are enriched within methylation and histone modification regions, suggesting a strong correlation between epigenetic marks and diseases, a pattern that some recent studies support [48-50]. In fact, cancer predisposing germline variants are over ten times more enriched within histone modification regions and chromatin physical interaction regions. There are no prominently enriched regulatory regions for complex disease variants, which present quite even enrichment distribution throughout all types of regulatory regions. Interestingly, complex disease variants show a positive enrichment within transcription enhancer, while other types of diseases variants have low negative enrichment, suggesting transcription enhancers might play an important role during complex disease development compared to other types of diseases. All four types of disease variants are enriched within DNA binding sites of protein by ChIP-seq, DNase I hypersensitive sites by DNase-seq, and open chromatin regions by FAIRE-seq. Disease-associated variants in different disease categories show dissimilar enrichment patterns within diverse regulatory elements, implying distinct priority of regulatory pathogenic mechanisms for different type of disease variants.

Considering that the majority of regulatory regions are located outside coding regions, and the distinct ratios of coding and noncoding disease variants in four types of disease categories may cause an acquisition bias on enrichment analysis, we further recalculated the enrichment analysis for only noncoding disease variants in four types of disease categories to eliminate the potential acquisition bias (Figure 3B, Table S8). By and large, the enrichment profile of noncoding disease variants is similar to that of all disease variants. Noncoding disease variants for Mendelian disease and cancer (germline or somatic) shows high enrichments within transcription promoter. Noncoding cancer germline variants are over ten times enriched within chromatin physical interaction regions. The highest enrichment within transcription enhancer is from complex disease variants. The outstanding enrichment difference between all disease variants and noncoding disease variants, occurs within histone modification regions, a dramatic decrease of enrichments, which conversely implies a tight association between histone modification epigenetic marks and disease variants that are located within coding regions. A recent study showed that histone modifications marks can be used to predict coding exon inclusion levels [51], which supports the idea that if the histone modification regions are altered by disease variants, then the change of target exons expression can be expected, potentially leading to disease formation. On the whole, noncoding disease variants and all disease variants show similar enrichment profiles within various regulatory regions.

**Figure 4:**
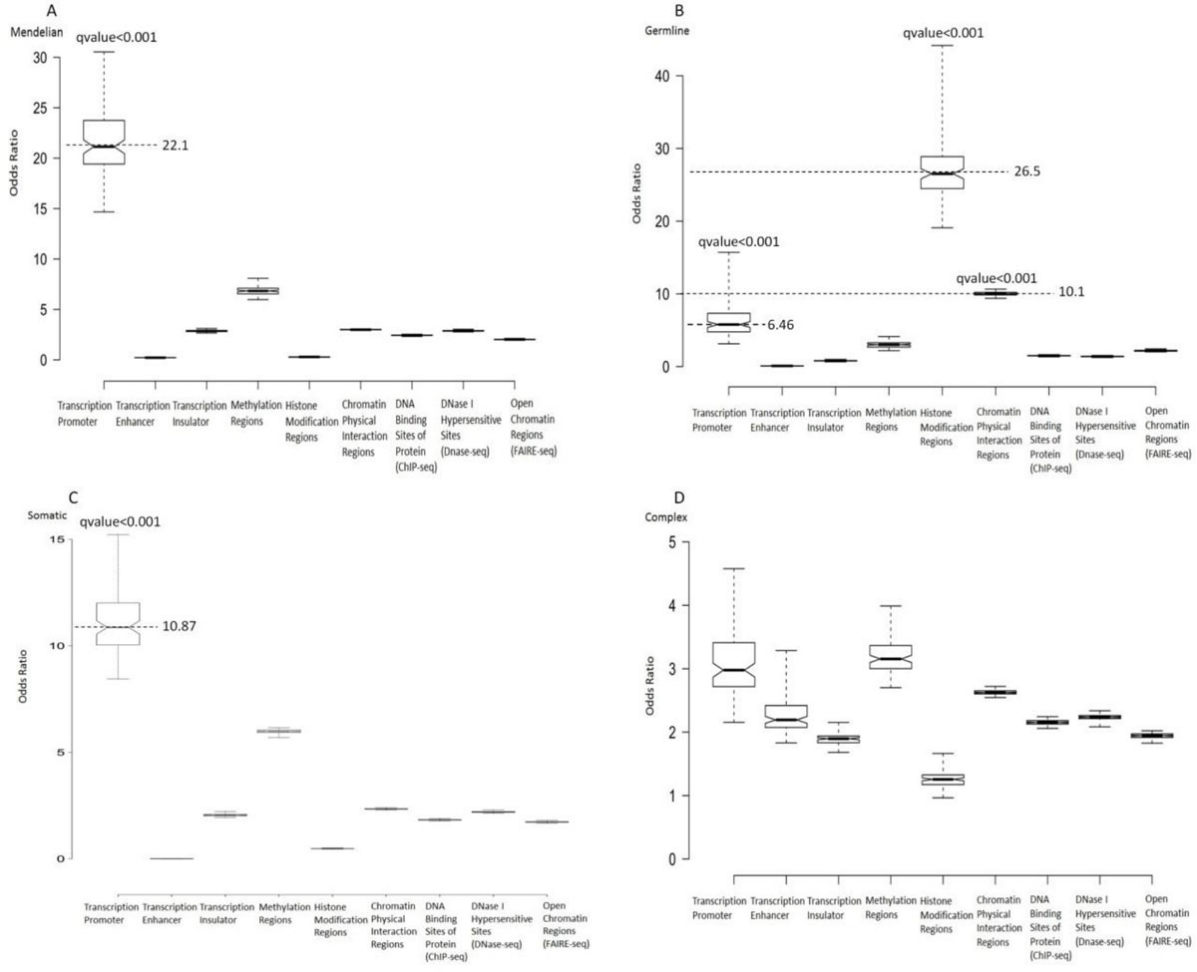
**Particular enriched regulatory regions for four type of disease variants**. For each type of disease variants, we generated 1000 equal size control groups according to the allele frequency distribution of disease variants, then calculated the odds ratio of disease variants to 1000 control groups respectively. Boxplots for (A) Mendelian disease variants, (B) cancer predisposing germline variants, (C) recurrent cancer somatic mutations and (D) complex disease variants. Transccription promoter are most enriched regulatory regions for Mendelian disease variants and recurrent cancer somatic variants. Cancer predisposing germline variants show high enrichemnt within histone modification regions, chromatin physical interaction regions and transcription promoter. No significant particular enriched regulatory regions for complex disease variants.

Different types of disease-associated variants show distinctive propensity for particular regulatory elements. We generated specific control groups for each type of disease variants to identify particular regulatory regions where disease-associated variants are enriched according to the following steps. Firstly, we generated the distribution of the allele frequencies for each type of disease variants based on the third phase of the 1000 genome project [52]. Secondly, for each type of disease variants, we randomly selected 1000 equal size control groups which share the same allele frequency distribution of disease variants. Next, for each type of disease variants, we calculated the odds ratios of disease variants to 1000 equal size specific control groups and statistically calculated each odds ratio using Pearson chi-squared test under p value threshold 0.05. Calculation of q value was based on the p values of 1000 enrichment analyses for each type of disease variant. The boxplot of the odds ratios for each type of disease variants are displayed in Figure 4. The Mendelian disease variants and recurrent cancer somatic mutations are most enriched in transcription promoter regions with median odds ratios of 22.1 and 10.87 respectively (Figure 4A,C). Cancer predisposing germline variants have a median odds ratio of 26.5 in histone modification regions, 10.1 in chromatin physical interaction regions, and 6.46 in transcription promoter respectively (Figure 4B). Complex disease variants have a quite even enrichment distribution within the various regulatory regions (Figure 4D). We further repeated such analysis for only noncoding disease variants (Figure S3). Noncoding disease variants for Mendelian disease and germline cancer are most enriched within transcription promoter with median odds ratios of 8.96 and 18.82 respectively (**Figure S3A, S3C**). A dramatic drop in enrichment occurs from all cancer predisposing germline variants to noncoding cancer predisposing germline variants (Figure 4B, **S3B**). Noncoding cancer predisposing germline variants present median odds ratio 1.2 within histone modification regions against 26.5 of all cancer predisposing germline variants. Noncoding cancer predisposing germline variants still show relatively high enrichments within chromatin physical interaction regions and transcription promoters with a median odds ratio of 3.04 and 2.34, respectively. Noncoding complex disease variants still present quite even enrichment distribution (**Figure S3D**). The analysis result for the complex disease variants replicated in at least two different ethnicities, further confirms that no particular enriched regulatory region for complex disease variants (Figure S4). The enrichment analysis for all disease variants or noncoding disease variants based on allele-frequency-matched genomic background, indicate the distinct particular enriched regulatory regions for different types of disease variants.

The two types of enrichment analyses of disease variants, based on dbSNP control group and 1000 equal size specific control groups, both suggest that disease-associated variants in different disease categories preferentially locate within particular regulatory regions.

## Conclusions

We curated 27,558 Mendelian disease variants, 20,964 complex disease variants, 5,809 cancer predisposing germline variants, and 43,364 recurrent cancer somatic mutations, and compared them against nine types of regulatory regions. Mendelian disease variants and recurrent cancer somatic mutations are 22- and 10-fold significantly enriched in promoter regions with q<0.001 respectively, compared to allele-frequency-matched genomic background. Different from these two categories, cancer predisposing germline variants are 27-fold enriched in histone modification regions (q<0.001), 10-fold enriched in chromatin physical interaction regions (q<0.001), and 6-fold enriched in transcription promoter (q<0.001). However, we observed a dramatic enrichment drop for noncoding cancer predisposing germline variants, with only 3-fold and 2-fold enrichment in chromatin physical interaction regions and transcription promoter regions with q<0.001, respectively. Furthermore, Mendelian disease variants and recurrent cancer somatic mutations share very similar distributions across types of functional impacts, suggesting the discovery of Mendelian disease variants might be broad enough to cover major pathways.

We also found that nine types of regulatory regions are located within over 50% of coding exon regions, suggesting the regulatory role of coding regions during gene expression. Transcription promoters, methylation regions, and transcription insulators have the highest density of disease variants, with 472, 239, and 72 disease variants per one million base pairs, respectively.

We recommend that different types of regulatory regions should be investigated for different categories of diseases, and the disease variants curated in this study provide a valuable resource for researchers to investigate the functional impact of disease variants.

## Methods

This study applied computational analytical methods to explore the pathogenic mechanism of disease-associated variants in different disease categories primarily at the regulatory level.

### Enrichment analysis

We compiled all disease-associated variants from multiple data sources. We subtracted disease-associated variants from all SNPs of dbSNP database and considered the remaining SNPs the genome variant background or control group. We then calculated the odds ratio of disease variants to human genome variant background within various regulatory elements. One thousand equal sized specific control groups were generated for each type of disease variants as a further validation experiment. Here we took Mendelian disease variants within promoter regions, for example, to detail how we calculated odds ratio. The Mendelian disease variants were collected from OMIM and ClinVar, and the promoter elements from the FANTOM5 project. The 2 × 2 contingency table (Table 5) shows the number of variants that locate within or outside promoter regions for Mendelian variants or control group SNPs. As such, the relative enrichment of Mendelian disease variants to the control group was measured by the resulting odds ratio, which is calculated by the following formula:

**Table 5:** 2 × 2 contingency table containing the number of Mendelian disease variants and control group SNPs located within or outside promoters for odds ratio calculation.

	Within promoter	Outside promoter
Mendelian disease	*DMW*	*DMO*
Control group	*DCW*	*DCO*

$$OR=\frac{D_{MW}/D_{MO}}{D_{CW}/D_{CO}}$$

We then calculate the natural logarithm of the odds ratio and the corresponding standard error. The standard error for the log odds ratio is calculated by the following formula:

$$SE=\sqrt{\frac{1}{D_{MW}}+\frac{1}{D_{MO}}+\frac{1}{D_{CW}}+\frac{1}{D_{CO}}}$$

Lastly, Pearson chi-squared test was performed on the 2 × 2 contingency table using a perl module Statistics::ChisqIndep from CPAN.

## Competing interests

The author declares that there is no conflict of interests in relation to this article.

## Authors' contributions

Rong Chen, Meng Ma, Ying Ru designed the study and prepared the manuscript. Jörg Hakenberg and Wei-Yi Cheng collected part of disease variants data and regulatory elements data. Meng Ma did the computational analysis. Ying Ru, Ling-Shiang Chuang, Nai-Yun Hsu and Li-Song Shi interpreted the analysis results. Andrew Uzilov, Wei Ding and Benjamin S. Glicksberg provided many valuable advices.

## Supplementary Material

Additional File 1 **Disease-associated variants for Mendelian diseases and complex diseases, and recurrent cancer somatic mutations**. Cancer predisposing germline variants can be downloaded from HGMD Professional. Click here for file

Additional File 2 Click here for file
