# Supplementary material for: Disease-associated variants in different categories of disease located in distinct regulatory elements
Source: BMC Genomics. 2015 Jun 18;16(Suppl 8):S3. doi: 10.1186/1471-2164-16-S8-S3 (PMC4480828; doi:10.1186/1471-2164-16-S8-S3)
Supplement: Additional File 2 [file 1471-2164-16-S8-S3-S2.pdf]

Supplementary Materials for

Disease-associated variants in different categories of disease located in distinct regulatory elements

Meng Ma[1,4#], Ying Ru[1,3#], Ling-Shiang Chuang[1], Nai-Yun Hsu[1], Li-Song Shi[1], Jörg Hakenberg[1], Wei-Yi Cheng[1], Andrew Uzilov[1], Wei Ding[1], Benjamin S. Glicksberg[1,2], Rong Chen[1]*
1Department of Genetics and Genomic Sciences, Icahn School of Medicine at Mount Sinai, New York, NY, 10029, USA
2Department of Neuroscience, Icahn School of Medicine at Mount Sinai, New York, NY, 10029, USA
3Department of Endocrinology, Anhui Provincial Hospital, Hefei, Anhui, 230001, China
4School of Computer Science and Technology, Anhui University, Hefei, Anhui, 230039, China

Email addresses for all authors
Meng Ma : meng.ma@mssm.edu
Ying Ru : ruying2@gmail.com
Ling-Shiang Chuang: ling-shiang.chuang@mssm.edu
Nai-Yun Hsu: nai-yun.hsu@mssm.edu
Li-Song Shi: lisong.shi@mssm.edu
Jörg Hakenberg : joerg.hakenberg@mssm.edu
Wei-Yi Cheng : wei-yi.cheng@mssm.edu
Andrew Uzilov : Andrew.uzilov@mssm.edu
Wei Ding : wei.ding@mssm.edu
Benjamin S. Glicksberg : Benjamin.glicksberg@mssm.edu
Rong Chen : rong.chen@mssm.edu

Corresponding author:
Rong Chen : rong.chen@mssm.edu

Co-First authors#:
Meng Ma: meng.ma@mssm.edu
Ying Ru: ruying2@gmail.com

Supplementary Text

Regulatory regions from FANTOM and ENCODE

This study focuses on the pathogenic mechanism study of disease-associated variants in different categories of disease at regulatory level. All datum used in this study are based on Human GRCH37/hg19. All variants and regulatory regions were extracted from multiple data sources before May 2014. We collected various regulatory regions from FANTOM5 and ENCODE project. Transcription promoter data of FANTOM5 can be downloaded from http://fantom.gsc.riken.jp/5/datafiles/latest/extra/CAGE_peaks/ (filename is hg19.cage_peak_ann.txt.gz); Transcription enhancer data of FANTOM5 can be downloaded from http://fantom.gsc.riken.jp/5/datafiles/latest/extra/Enhancers/ (filename is hg19_enhancers.bed.gz) . We downloaded regulatory regions identified by the ENCODE project through *Table Browser* of UCSC (http://genome.ucsc.edu) and the detailed information are in the following. Regulatory regions identified by DNase-seq was download from "DNase Clusters" track (table name is wgEncodeRegDnaseClusteredV2). DNA Binding sites of proteins by ChIP-seq were downloaded from "Txn Factor ChIP" track (table name is wgEncodeRegTfbsClusteredV3). Insulator regions are the binding sites of CTCF, which were also extracted from "Txn Factor ChIP" track. Regulatory regions identified by FAIRE-seq were downloaded from "UNC FAIRE" track in 36 cell lines. Chromatin physical interaction regions by CHIA-PET were downloaded from "GIS ChIA-PET" track in five different human cancer cell lines. Histone modification regions were downloaded from "Broad Histone" track. CpG methylation regions by Methyl 450K Bead Arrays were downloaded from "HAIB Methyl450" with score>200.

Disease-associated Variants

We collected four types of disease-associated variants including Mendelian disease variants, complex disease variants, cancer predisposing germline mutations, and recurrent cancer somatic mutations. We counted the number of associated diseases/phenotypes/traits and the genes where disease variants are located within. All transcripts of the genes are extracted from UCSC "GENCODE Genes V19" track (filename is wgEncodeGencodeBasicV19). We extended each transcript with the flanking 500bp. Mendelian disease variants were gotten from OMIM and ClinVar. We downloaded and parsed a compressed file of OMIM site from http://www.omim.org/downloads (filename is omim.txt.Z) and extracted all OMIM disease variants. All pathogenic variants of ClinVar can be obtained from ftp://ftp.ncbi.nlm.nih.gov/pub/clinvar/tab_delimited/ (filename is variant_summary.txt.gz). We collected 27,558 Mendelian disease variants finally. Complex disease variants were obtained from NHGRI GWAS catalog (http://www.genome.gov/admin/gwascatalog.txt) and VarDi. VarDi is a proprietary

database of disease-associated variants built through a combination of Hadoop-based text mining tools and manual curation. We collected 20,964 complex disease variants under p value < 10E-8. Considering the fact that most complex disease variants are mark SNPs, and not necessarily the disease causal variants, we filtered complex disease variants using stricter criterion. We accept such hypothesis that if a complex disease SNP is replicated in different ethnicities, then this SNP is more likely to be causal than markers. Those complex disease variants replicated in at least two different ethnicities, were adopted as complex disease causal variants. At last 5,549 complex disease causal variants were collected. Cancer germline mutations were downloaded from HGMD Professional database (http://www.biobase-international.com/product/hgmd). We collected 5,809 cancer predisposing germline variants. Recurrent cancer somatic mutations were obtained from COSMIC (https://cancer.sanger.ac.uk/files/cosmic/ , filename is CosmicCompleteexport_v68_040214). Only mutations which are with "Confirmed somatic variant" status, and recurrent in at least two different samples, will be adopted. At last 43,364 recurrent cancer somatic mutations were collected. All SNPs of dbSNP137 were downloaded from "ALL SNPs(137)" track of UCSC (table name is snp137) at June 2013. We collected 53,557,889 SNPs as human genome variant background.

Exome DNA sequencing technologies

Illumine SureSelect TruSeq and Nimblegene SeqCap EZ are two popular exome DNA sequencing platforms to identify Mendelian disease variants, cancer predisposing germline mutations or cancer somatic mutations. The target regions of Illumine SureSelect TruSeq is downloaded from http://support.illumina.com/sequencing/sequencing_kits/truseq_exome_enrichment_kit/downloads.html. The target regions of Nimblegene SeqCap EZ is downloaded from http://www.nimblegen.com/products/seqcap/ez/v3/index.html.

Regulatory regions can locate within coding and noncoding regions

We extracted upstream and downstream 2000bp of genes, coding exons, 5' and 3' UTR, and introns, and intergenic regions through "GENCODE Genes V19" track (filename is wgEncodeGencodeBasicV19). Then we counted the length of human genome regions overlapping with nine types of regulatory regions using BEDTools utilities *Intersect* and *merge* (https://code.google.com/p/bedtools/).

Functional effect annotation of disease-associated variants

There are 34 consequences of genetic variants in Sequences Ontology (http://www.sequenceontology.org/), and Ensembl rank these consequences in the order of severity. This ordering is necessarily subjective. Everyone may always extract the full set of consequences for each allele and make their own severity judgment. We applied Ensembl Variant Effect Predictor web interface

(http://grch37.ensembl.org/Homo_sapiens/Tools/VEP) to annotate the four types of disease-associated variants based on Ensembl transcripts.

Predicting scores for disease-associated variants

Genome Wide Annotation of Variants (GWAVA) is from the Wellcome Trust Sanger Institute and the European Bioinformatics Institute. GWAVA aims to predict functionality of noncoding variants based on a wide range of annotations of regulatory elements, along with genome-wide properties such as evolutionary conservation and GC-content. Corresponding with three different control groups adopted in GWAVA, there are three kinds GWAVA scores: Region score, TSS score and Unmatched score, which are all in the range of 0-1. A high GWAVA score means more active functionality with respect to a low GWAVA score. . The GWAVA score can be computed in this website https://www.sanger.ac.uk/sanger/StatGen_Gwava.

Mutation Assessor predicts the functional impact of coding variants on proteins based on sequence evolutionary conservation. Usually functional coding variants have higher Mutation Assessor score than non-functional coding variants. Mutation Assessor score threshold of 1.9 was used to discriminate disease-associated variants with medium or high functionality; if the Mutation Assessor score of a variants is smaller than 1.9, then this variant can be considered to be low functional or neutral. The Mutation Assessor score can be computed in this website http://mutationassessor.org/.

Combined Annotation-Dependent Depletion (CADD) is from the University of Washington and HudsonAlpha Institute for Biotechnology. CADD framework incorporates a support vector machine in its workflow. CADD integrates multiple annotations into one metric by contrasting variants that survived natural selection with simulated mutations in order to score the deleteriousness of coding or noncoding variants in human genome. A high CADD score typically suggests more severe deleteriousness compared to a low CADD score. There are two distinct CADD score forms, namely “Raw” and “Scaled”. “Raw” CADD scores come straight from the SVM and have relative meaning, with higher values indicating that a variant is more likely to have deleterious effects. “Scaled” score is as transformation of “Raw” score by ranking all variants. For example, reference genome single nucleotide variants at the top 10% of CADD scores are assigned to CADD-10. In this study, we adopted CADD “Raw” score. Pre-computed CADD score of all possible SNVs of hg19 can be downloaded from http://cadd.gs.washington.edu/download (filename is whole_genome_SNVs.tsv.gz).

GERP produces position-specific estimates of evolutionary constraint using maximum likelihood evolutionary rate estimation. Negative GERP scores indicate that a site is probably evolving neutrally. Positive scores indicate that a site may be under

evolutionary constraint. Positive scores scale with the level of constraint, such that the greater the score, the greater the level of evolutionary constraint. GERP code can be downloaded from http://mendel.stanford.edu/SidowLab/downloads/gerp/.

Enrichment analysis of disease-associated variants within regulatory regions

The enrichment of disease-associated variants can be measured using odds ratio (see Methods section).

Two different strategies about control group generation were adopted for enrichment analysis of disease-associated variants within regulatory regions.

The first strategy is to make the human genetic variant background as control group for each type of disease-associated variants. We subtracted disease-associated variants from all SNPs of dbSNP database and made remain SNPs as the genome variant background control group.

The second strategy is to generate 1000 equal size specific control groups for each type of disease-associated variants. Based on the allele frequency distribution of each type of disease-associated variants, we generated 1000 equal size control groups for disease variants.

Figure S1

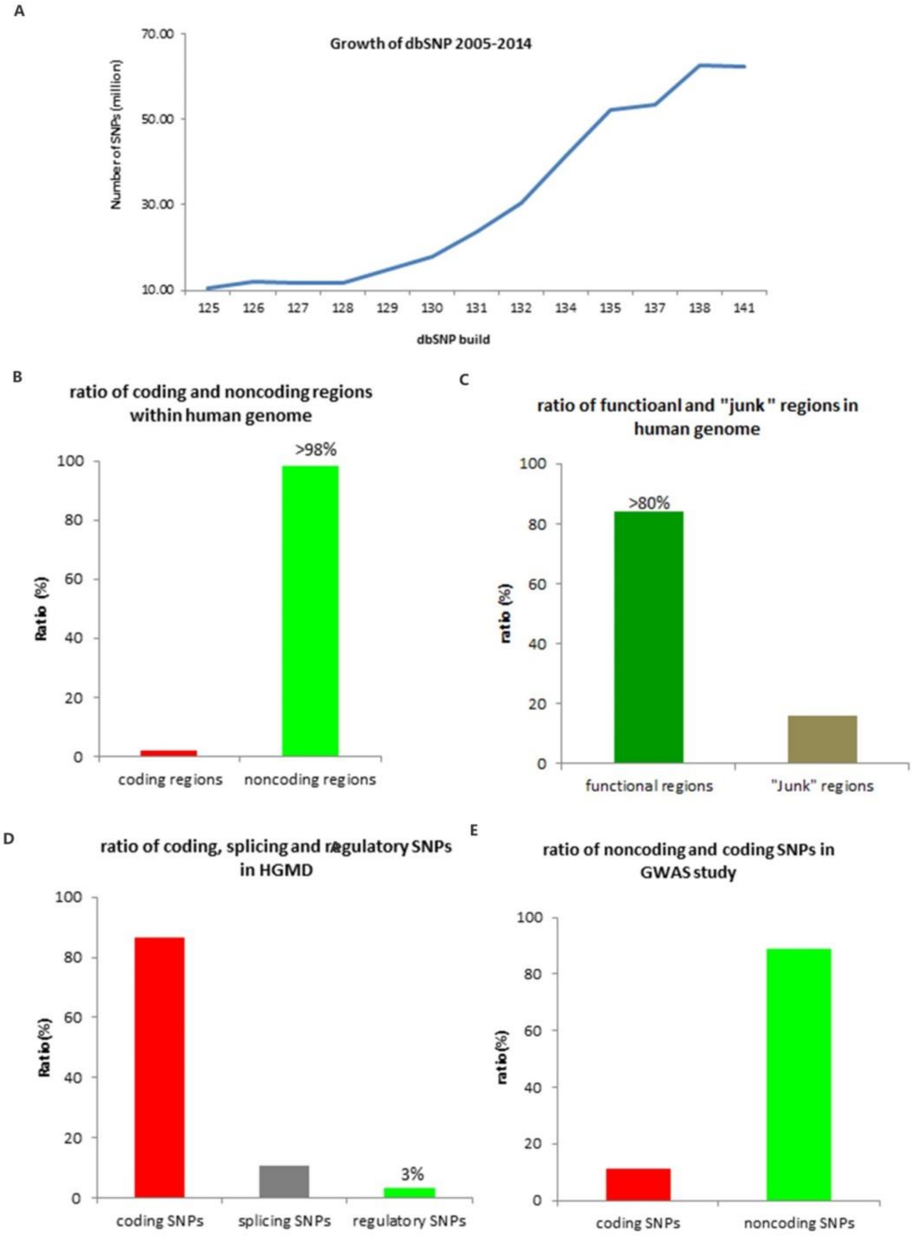

Figure S1. Human genomic variants. (A) Dramatic growth of dbSNP along with wide application of high throughput sequencing technology. (B) Over 98% of human genome is noncoding region. (C) ENCODE study show that over 80% of human genome is functional. (D) Most well annotated disease variants are coding variants. (E) GWAS study show that ~90% of complex disease variants are noncoding variants.

Figure S2

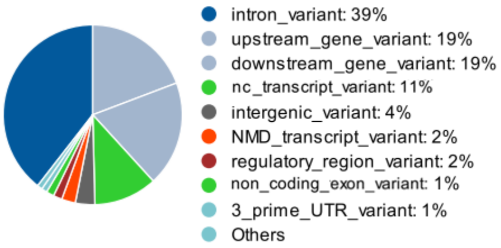

Figure S2. Functional effect annotation of complex disease variants that are replicated in at least two different ethnicities. The majority of complex disease variants are noncoding variants.

Figure S3

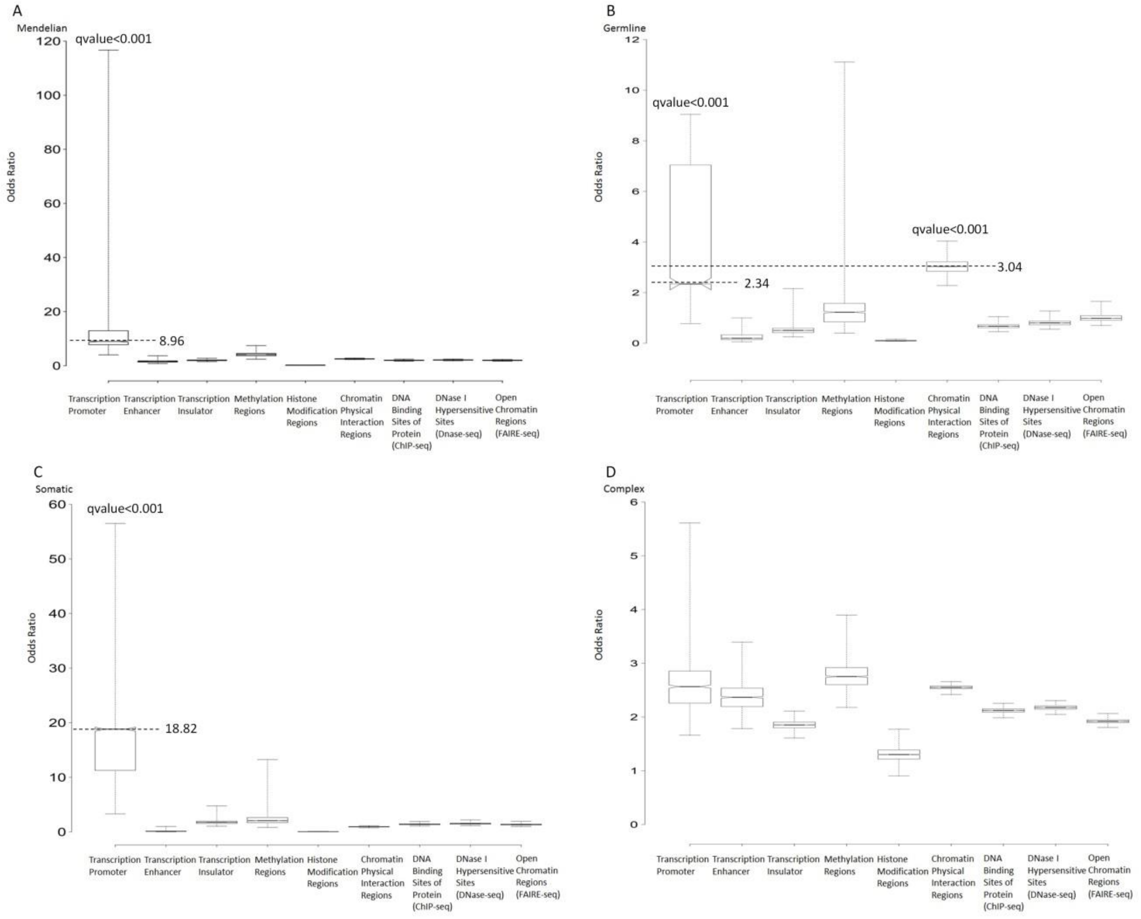

Figure S3. Particular enriched regulatory regions for four type of noncoding disease variants. For each type of noncoding disease variants, we did enrichment analysis based on 1000 equal size control groups generated according to the allele frequency distribution of noncoding disease variants. Boxplots for (A) Mendelian disease noncoding variants, (B) cancer predisposing germline noncoding variants, (C) recurrent cancer somatic noncoding mutations and (D) complex disease noncoding variants. Mendelian disease noncoding variants and recurrent cancer somatic noncoding variants show the highest enrichment within transcription promoter. Cancer predisposing germline noncoding variants are enriched within chromatin physical interaction regions and transcription promoter. Complex disease noncoding variants doesn't show particular enriched regulatory regions.

Figure S4

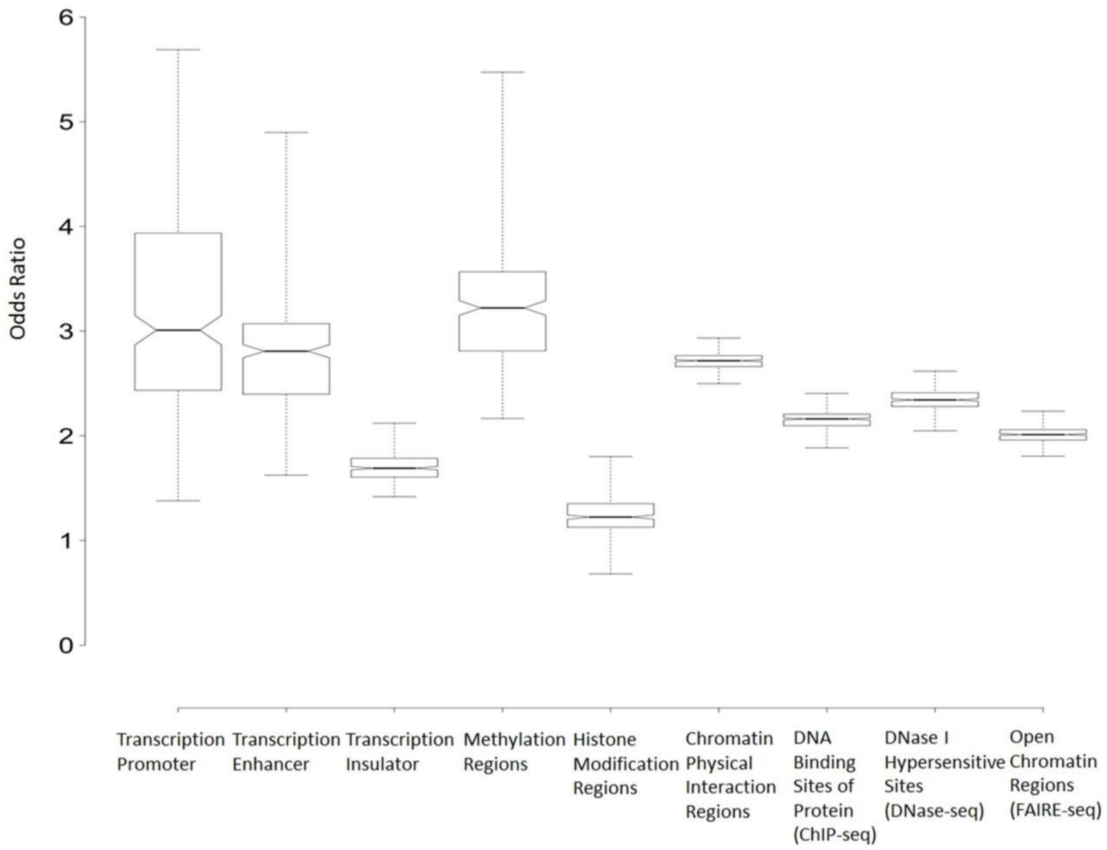

Figure S4. 1000 times enrichment anlaysis for complex diseasae variants from multiple ethnicities based on allele-frequency-matched genomic background. No particular enriched regulatory regions for complex disease noncoding variants.

Table S1. Percentage of each type of human genomic regions overlapped with different regulatory regions

	Promoter (%)	Enhancer (%)	Insulator (%)	Methylation regions (%)	Histone modification regions (%)	Chromatin physical interaction regions (%)	DNA binding sites of protein (%)	Open chromatin regions (DNase I hypersensitive sites) (%)	Open chromatin regions by FAIRE-seq (%)	Count (%)
Coding Exons (36,106,161bp)	0.88	0.0008	4.65	2.66	54.94	40.12	14.55	17.47	13.56	54.99
Upstream (117,214,781bp)	0.88	0.38	5.6	2.62	55.7	39.1	19	18.81	15.46	55.80
3'-UTR (46,842,296bp)	0.5	0.03	3.51	1.69	59.12	42.84	13.99	14.87	13.02	59.17
5'-UTR (23,784,833bp)	3.34	0.035	8.44	3.58	55.75	40.82	23.41	23.65	19.39	55.83
Introns (1,413,709,196bp)	0.067	0.31	1.77	0.39	63.75	32.86	9.23	9.32	9.7	63.80
Downstream (123,496,320bp)	0.16	0.26	2.68	0.84	56.02	37.31	12.1	12.15	10.3	56.11
Intergenic region (1,873,616,100bp)	0.09	0.41	2.64	0.57	97.18	40.65	11.99	12.24	15.5	98.32

Table S2. Percentage of Illumina SureSelect TruSeq regions and Nimblegen SeqCap_EZ_Exome_v3 regions overlapped with various human genomic regions

	Upstream (%)	5'-UTR (%)	Coding Exon (%)	Introns (%)	3'-UTR (%)	Downstream (%)	Intergenic Regions (%)
TruSeq Regions (62,085,295bp)	11.15	15.16	51.54	22.73	43.16	24.77	0
SeqCap Regions (63,564,965bp)	10.03	10.12	53.75	43.95	16.14	13.65	0

Table S3. Percentage of human genomic regions overlapped with Illumina SureSelect TruSeq regions and Nimblegen SeqCap_EZ_Exome_v3 regions

	**TruSeq Regions (%)**	**SeqCap Regions (%)**
Coding Exons (36,106,161bp)	88.62	94.63
Upstream (117,214,781bp)	5.91	5.44
3'-UTR (46,842,296bp)	57.20	21.90
5'-UTR (23,784,833bp)	39.57	27.05
Introns (1,413,709,196bp)	0.99	1.98
Downstream (123,496,320bp)	12.45	7.03
Intergenic region (1,873,616,100bp)	0	0

Table S4. Percentage of Illumina SureSelect TruSeq regions and Nimblegen SeqCap_EZ_Exome_v3 regions overlapped with various regulatory regions

	Promoter (%)	Enhancer (%)	Insulator (%)	Methylation regions (%)	Histone modification regions (%)	Chromatin physical interaction regions (%)	DNA binding sites of protein (%)	Open chromatin regions (DNase I hypersensitive sites) (%)	Open chromatin regions by FAIRE-seq (%)
TruSeq Regions (62,085,295bp)	2.51	0.0047	9.21	5.22	99.34	75.90	29.40	33.19	27.30
SeqCap Regions (63,564,965bp)	1.57	0.044	8.22	4.47	97.43	72.18	26.63	31.59	25.62

Table S5. Percentage of various regulatory regions overlapped with Illumina SureSelect TruSeq regions and Nimblegen SeqCap_EZ_Exome_v3 regions

	**TruSeq Regions (%)**	**SeqCap Regions (%)**
Promoter (3,833,500bp)	40.65	26.03
Enhancer (12,385,403 bp)	0.02	0.23
Insulator (81,713,060 bp)	6.99	6.39
Methylation region (19,517,834 bp)	16.60	14.56
Histone modification region (2,816,878,674 bp)	2.19	2.19
Chromatin physical interaction regions (1,288,430,643 bp)	3.66	3.56
DNA binding sites of protein (380,355,257 bp)	4.80	4.45
Open chromatin regions (DNase I hypersensitive sites) (387,138,495 bp)	5.32	5.19
Open chromatin regions by FAIRE-seq (448,557,442 bp)	3.78	3.63

Table S6. Consequences of genomic variants from Sequence Ontology which are ranked in the order of severity by Ensembl analysis group

Consequences	Description
transcript_ablation	A feature ablation whereby the deleted region includes a transcript feature
splice_donor_variant	A splice variant that changes the 2 base region at the 5' end of an intron
splice_acceptor_variant	A splice variant that changes the 2 base region at the 3' end of an intron
stop_gained	A sequence variant whereby at least one base of a codon is changed, resulting in a premature stop codon, leading to a shortened transcript
frameshift_variant	A sequence variant which causes a disruption of the translational reading frame, because the number of nucleotides inserted or deleted is not a multiple of three
stop_lost	A sequence variant where at least one base of the terminator codon (stop) is changed, resulting in an elongated transcript
initiator_codon_variant	A codon variant that changes at least one base of the first codon of a transcript
transcript_amplification	A feature amplification of a region containing a transcript
inframe_insertion	An inframe non synonymous variant that inserts bases into in the coding sequence
inframe_deletion	An inframe non synonymous variant that deletes bases from the coding sequence
missense_variant	A sequence variant, that changes one or more bases, resulting in a different amino acid sequence but where the length is preserved
splice_region_variant	A sequence variant in which a change has occurred within the region of the splice site, either within 1-3 bases of the exon or 3-8 bases of the intron
incomplete_terminal_codon_variant	A sequence variant where at least one base of the final codon of an incompletely annotated transcript is changed
feature_truncation	A sequence variant that causes the reduction of a genomic feature, with regard to the reference sequence
stop_retained_variant	A sequence variant where at least one base in the terminator codon is changed, but the terminator remains
synonymous_variant	A sequence variant where there is no resulting change to the encoded amino acid
coding_sequence_variant	A sequence variant that changes the coding sequence
mature_miRNA_variant	A transcript variant located with the sequence of the mature miRNA
5_prime_UTR_variant	A UTR variant of the 5' UTR
3_prime_UTR_variant	A UTR variant of the 3' UTR
non_coding_exon_variant	A sequence variant that changes non-coding exon sequence
intron_variant	A transcript variant occurring within an intron
NMD_transcript_variant	A variant in a transcript that is the target of NMD
nc_transcript_variant	A transcript variant of a noncoding RNA
upstream_gene_variant	A sequence variant located 5' of a gene
downstream_gene_variant	A sequence variant located 3' of a gene
TFBS_ablation	A feature ablation whereby the deleted region includes a transcription factor binding site
TFBS_amplification	A feature amplification of a region containing a transcription factor binding site
TF_binding_site_variant	A sequence variant located within a transcription factor binding site
regulatory_region_ablation	A feature ablation whereby the deleted region includes a regulatory region
regulatory_region_amplification	A feature amplification of a region containing a regulatory region
regulatory_region_variant	A sequence variant located within a regulatory region
feature_elongation	A sequence variant that causes the extension of a genomic feature, with regard to the reference sequence
intergenic_variant	A sequence variant located in the intergenic region, between genes

Table S7. P values by Pearson chi-squared test for the disease-associated variants and the SNPs from genomic variants background control group within or outside regulatory regions.

	Mendelian disease	Cancer Germline	Cancer Somatic	Complex disease
Transcription Promoter	0	1.2666E-53	0	2.2932E-12
Transcription Enhancers	6.4174E-16	0.10327E-4	0	1.3567E-33
Transcription Insulator	0	0.20549	0	0
Methylation Regions	0	6.7005E-57	0	0
Histone Modification Regions	3.1527E-20	7.5703E-08	1.5E-10	1.3777E-15
Chromatin Physical Interaction Regions	0	0	0	0
DNA Binding Sites of Protein (ChIP-seq)	0	4.2317E-40	0	0
DNase I Hypersensitive Sites	0	7.555E-31	0	0
Open Chromatin Regions (FAIRE-seq)	0	0	0	0

Table S8. P values by Pearson chi-squared test for the noncoding disease-associated variants and the noncoding SNPs from genomic variants background control group within or outside regulatory regions.

	Mendelian disease	Cancer Germline	Cancer Somatic	Complex disease
Transcription Promoter	0	2.8307E-06	0	3.3004E-20
Transcription Enhancers	0.027013	0.24207	0.053474	2.8546E-35
Transcription Insulator	1.8242E-28	0.19099	4.9963E-20	0
Methylation Regions	0	0.055422	2.7062E-15	0
Histone Modification Regions	0	0.40436	1.0792E-21	0.00037287
Chromatin Physical Interaction Regions	0	0	8.1375E-18	0
DNA Binding Sites of Protein (ChIP-seq)	0	0.24175	1.6206E-26	0
DNase I Hypersensitive Sites	0	0.93314	3.6857E-37	0
Open Chromatin Regions (FAIRE-seq)	0	0.023574	2.0194E-19	0
